# Prostate Rhabdomyosarcoma presenting as acute urinary retention in a young adult

**DOI:** 10.1016/j.eucr.2022.102083

**Published:** 2022-04-20

**Authors:** Ana Domínguez, Javier Lorca, David López-Curtis, Vital Hevia, Enrique Sanz, Francisco Javier Burgos Revilla

**Affiliations:** Hospital Universitario Ramón y Cajal. Urology Department, Hospital Inversitario Ramón y Cajal, Madrid, Spain

**Keywords:** Prostate, Rhadomyosarcoma, Conservative surgery

## 1 Introduction

Rhabdomyosarcoma (RMS) is a mesenchymal tumor, common in pediatric age but unusual in adults and adolescents. It represents 10–15% of all malignant solid tumors in children.^1^ 5–10% of them are located in the urinary system, mainly in the prostate and bladder (BP-RMS).^2^ It is considered a type of prostate cancer with high degree of aggressiveness, local invasion and metastatic disease.

Acute urinary retention is an uncommon urinary symptom in young adults and it's mainly due to benign pathologies, such as urethral stricture. However, prostate RMS can be presented as voiding symptoms and urinary obstruction, so it must be included in the differential diagnosis.

Early and multidisciplinary treatment of BP-RMS is mandatory. One of the main goals of treatment in children and young adults is to preserve the urinary bladder function to achieve a good quality of life in these patients. In an attempt to preserve these organs without affecting overall survival, a conservative approach based on polychemotherapy followed by radiotherapy or/and bladder-sparing surgery can be performed in some cases.^3^, ^4^, ^5^

## 2 Case report

An 18-year-old healthy man attended to Emergency Department with acute urinary retention. He referred to a previous history of disuria without hematuria or other symptoms. Digital rectal examination showed and indurated mass, while laboratory blood and urinary test, as well as prostate specific antigen (PSA) levels were normal.

Ultrasonography (US) and computed tomography (CT) scan showed a highly enlarged heterogeneous prostate of 61 × 65 × 74 mm (volume of 152 cc) with a poorly defined polypod heterogeneous mass with a maximum axis of 71mm (Picture 1)*.*

**Picture 1:**
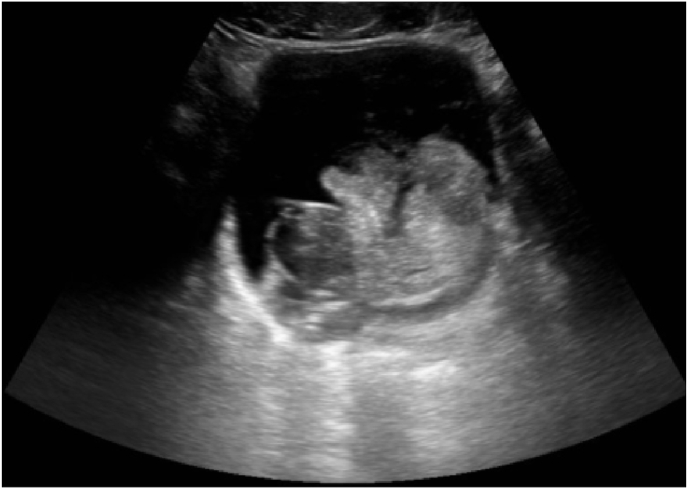
Prostate heterogeneous mass showed in US.

Multiparametric magnetic resonance (MRI) revealed a heterogeneous enhancing mass replacing the prostate with infiltration to the right lateral wall of the bladder, ischiocavernosus muscles and intersphincteric space, respecting the internal and external anal sphincter. There were no lymph nodes involved (Picture 2).

**Picture 2:**
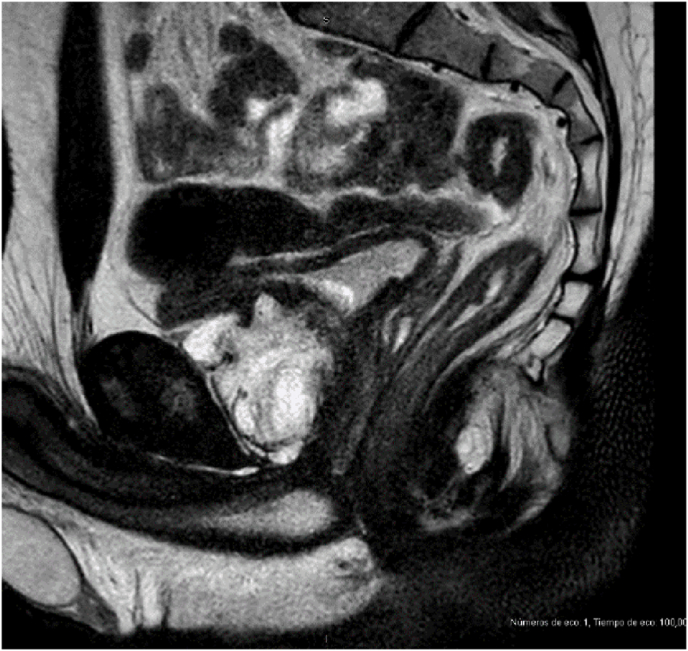
Prostate mass showed in MRI.

The patient underwent transurethral resection of the prostate (TURP) and the histopathology showed fragments of an intensely necrotic neoplasm with histological characteristics of embryonal RMS (Picture 3). On the immunohistochemistry study, the tissue specimen was positive for desmin and Myo D-1 with a Ki67 proliferation index of 80%.

**Picture 3:**
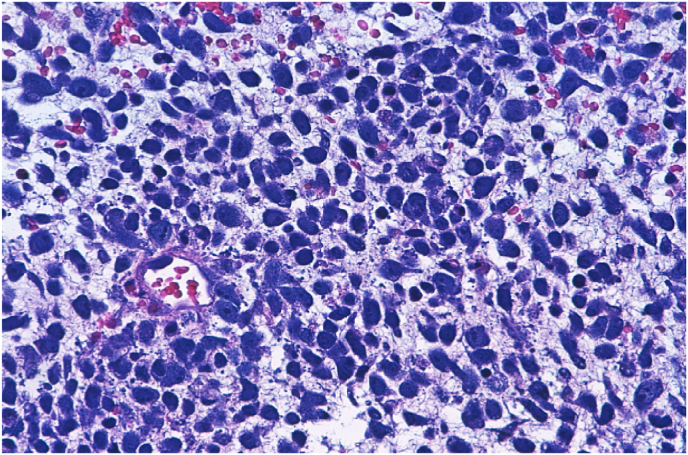
Histopathological tissue under microscope.

After pathological diagnosis, CT body was performed and no metastatic disease was found. According to a multidisciplinary committee's decision, the patient underwent induction chemotherapy based on 6 cycles of vincristine, actinomycin D and cyclophosphamide (VAC). Before VAC chemotherapy initiation, semen cryopreservation was performed. CT scan after the treatment showed a partial response so the patient maintained systematic treatment with chemotherapy adding local radiotherapy, followed by an intensification therapy with cyclophosphamide and vincristine (VC). However, in subsequents control CT and MRI there were no changes in the mass, so the patient finally underwent surgery, performing an uneventful radical cystoprostatectomy with ileal conduit as urinary diversion. Final specimen histopathology showed necrosis and fibrosis with absence of residual disease as well as free surgical margins. Finally, due to the aggressiveness of this type of tumor, the patient received adjuvant chemotherapy with 4 cycles of VAC and after a 2-year follow up he is currently free of disease.

## 3 Discussion

BP-RMS treatment is a real challenge, especially in childhood and young adults. Multimodal treatment protocols (based on chemotherapy, radiotherapy and surgery) have been developed -over the years-by several cooperative groups to establish treatment strategies. An improvement in the 5-year overall survival rates for BP-RMS has been observed due to the application of these multimodal protocols, increasing estimated survival from 25% in the 1970s to greater than 70% nowadays^2^

One of the main goals in BP- RMS management in children and young adults is to preserve urinary function without compromising overall survival, so treatment must be carefully individualized according to the tumors histological features, location, extent and pathological stage.

Based on these premises, two main protocols can be highlighted: the European according to the International Society for Pediatric Urology (SIOP) and the American according to the Children's Oncology Group (COG) guidelines.^4^^,^^5^ European approach protocol is based on delayed surgical excision after induction polychemotherapy, with the aim of reducing the tumors size and to aim for a less aggressive surgery. However, the American approach diverges in the main treatment to achieve local control of the disease, considering radiotherapy (after induction chemotherapy) the gold standard treatment to preserve the organ. Radical surgery is reserved to selected cases after radiotherapy relapse with the aim to resect residual disease with negative margins. Regardless of the approach, overall survival seems to be similar for BP-RMS (80% 5-year OS in SIOP vs 86% 5-year OS in COG).^5^

In recent years, brachytherapy and proton beam therapy have been added to the multimodal treatment for local control strategy to reduce morbidity associated with radiotherapy.^5^ Although optimal results have been obtained, the existing data is limited to establish changes in local control treatment.

There are several options for urinary reconstruction after cystoprostatectomy. It is important to choose the best option according to the patient's age, tumors aggressiveness, location and extension and surgeon's experience. After radical surgery, the most frequently urinary reconstruction performed is an incontinent diversion with ileal conduit.^2^, ^5^ Continent diversion can be performed by experienced surgeons once the patient has demonstrated durable survival from the disease.

Because BP RMS is an unusual and aggressive tumor in the young adult population, there are not enough studies assessing the long-term results regarding different urinary diversion types.

## 4 Conclussion

Although BP-RMS is an unusual tumor, it must be suspected in young adults with acute urinary retention. Early and multidisciplinary treatment is mandatory to achieve a good quality of life without compromising overall survival.

## Declaration of competing interest

The authors declare no conflict of interest.
